# Vibrational Molecular Spectroscopy as a Tool to Study Molecular Structure Features of Cool-Season Chickpeas Impacted by Varieties and Thermal Processing in Relation to Nutrient Availability in Ruminants

**DOI:** 10.3390/ani13020304

**Published:** 2023-01-15

**Authors:** Linda Cerna, María E. Rodríguez Espinosa, Weixian Zhang, Peiqiang Yu

**Affiliations:** 1 Department of Animal and Poultry Science, College of Agriculture and Bioresources, University of Saskatchewan, 51 Campus Drive, Saskatoon, SK S7N 5A8, Canada; 2 College of Animal Science and Technology, Henan University of Animal Husbandry and Economy, Zhengzhou 450046, China

**Keywords:** chickpeas, processing, FTIR, mid-infrared, spectroscopy

## Abstract

**Simple Summary:**

Feed molecular structure profile affects nutrient metabolism, utilization, and availability. Feed processing often induces feed internal structure change. These internal structure changes will affect animal nutrition. This study aimed to reveal the molecular structure features among chickpea varieties and detect the molecular structure changes induced by thermal processing methods. Our results show that with vibrational molecular spectroscopy, chickpea structure on a molecular basis was revealed in relation to ruminant nutrition.

**Abstract:**

To our knowledge, there is no study on the relationship between molecular spectral features and nutrient availability in chickpeas. The purpose of this study was to reveal molecular structure spectral profiles among cool-season adapted CDC chickpea varieties and detect the molecular structure changes induced by thermal processing methods using vibrational Fourier-transform infrared (FTIR) spectroscopy. Three varieties of chickpea samples (CDC Alma, Cory, Frontier) were finely ground using a 0.12 mm screen. Spectral analyses were conducted using a JASCO FTIR-4200 spectroscope with Spectra Manager II software in the mid-infrared region from ca. 4000–800 cm^−1^ with a 4 cm^−1^ resolution. Data were analyzed using the “Mixed” procedure of SAS 9.4. Multiple regression was performed with PROC REG analysis for variable selection. Results showed that amide I area was higher (*p* = 0.038) in CDC Frontier than CDC Cory (30.85 vs. 24.64 AU). Amide I peak height (*p* = 0.028) was also higher in CDC Frontier and CDC Alma (0.45 AU in both) than CDC Cory (0.36 AU). Cellulosic compound (CEC) to total CHO (TCHO) area ratio was higher in CDC Frontier (0.05 AU) than the other two varieties (0.14 AU in both). As to thermal treatment impact, the results showed that total amide area was higher (*p* = 0.013) with autoclave and microwave heating (47.38 and 45.19 AU, respectively) than dry heating (33.06 AU). The CEC area was also higher (*p* < 0.001) for autoclave and microwave heating (3.74 and 3.61 AU, respectively) than dry heating (2.20 AU). Moreover, the ratio of amide I to II height was higher (*p* = 0.022) with microwave heating than dry heating (1.44 vs. 1.16 AU, respectively). Relationship analysis showed that the effective degraded crude protein (EDCP) and bypass dry matter (% BDM) were associated with STCHO peaks and CEC height (*p* < 0.05, R^2^ = 0.68). Also, feed milk value (FMV_DVE_) was associated with STC1, STC_A, and CEC_A (*p* < 0.05, R^2^ = 0.85). In conclusion, vibrational molecular spectroscopy mid-infrared FTIR was able to reveal different molecular spectral characteristics among the cool-season adapted CDC chickpea varieties and detect molecular structure changes induced by thermal processing (dry heating, autoclaving, and microwave heating).

## 1. Introduction

Infrared (IR) spectroscopy has been successfully used as an analytical technique in organic chemistry since the 1940s. It has been widely applied in the analysis of chemical composition as a rapid and simple operation to determine multinutrient conformation in a nondestructive and nonpollutive manner [1]. The basic principle of IR spectroscopy is that following exposure to IR radiation, chemical functional groups exhibit specific energy absorptions at certain frequencies, which enables the detection of chemical and structural differences in a variety of samples. The typical IR absorption peaks of the relevant biopolymers have been well documented [2].

One common method based on IR analysis includes vibrational Fourier-transform infrared (FTIR) spectroscopy. This well-established experimental technique is used to study structural composition, stability, and conformational changes, such as the effects of temperature, pH, and pressure in feed materials [3]. The mathematical method of Fourier transform converts the symmetric interferogram into functions with frequency components to form continuous transmittance or absorbance spectra [4]. In comparison with conventional dispersive spectroscopy, FTIR spectroscopy exhibits more effective and powerful properties due to its excellent sensitivity, larger optical throughput, and good signal-to-noise (S/N) ratio. Some advantages of this technique are the rapid scan speed and the ease of detecting the entire IR region simultaneously [4].

Several chemical functional groups can be detected using FTIR spectroscopy. For instance, protein molecular structure is unique in its peptide bonds, which contain C=O, C–N, and N–H functional groups. Amide-related groups show energy absorbance peaks at around 1700–1500 cm^−1^. The amide I band is often used for protein structure analysis, as amide II usually overlaps other bands such as lignin at ca. 1515 cm^−1^ [5]. On the other hand, carbohydrate’s molecular structure has many OH and CO bonds. Some infrared spectra related to carbohydrates appear at ca. 1200–800 cm^−1^. Cellulose is mainly characterized around ca 1170–1150, 1050, and 1030 cm^−1^ and hemicellulose could be found at ca. 1732 and 1240 cm^−1^ [5,6]. Data obtained from spectral analysis lets researchers compute spectral ratios that represent the biological component ratio intensity and its distribution in the tissue. These ratios are obtained dividing the spectral height or area under one chemical functional group band (e.g., amide I) by the height or area under another functional group band (e.g., amide II) [7].

It is well stablished that thermal treatments inactivate antinutritional factors in legume seeds and improve their nutritional value. Additionally, heat processing alters not only the physical but also the intrinsic structures of feeds by (1) disrupting the protein configuration, making it more susceptible to digestive enzymes, (2) disrupting starch crystallites and promoting starch chain interactions within amorphous and crystalline areas, and (3) affecting hydration properties of fiber and modifying its physical properties [8]. As a result, these changes directly affect the functional properties, digestion, and absorption of several feeds.

The study of molecular structure characteristics of feedstuffs is hence important to stablish precise feeding techniques for ruminant systems. In our case, there is no systematic analysis on the relationship between molecular structure features of chickpeas and nutrient utilization and availability in ruminants. There is no study on the possible molecular structure changes induced by thermal processing in cool-season adapted chickpea varieties by using advanced vibrational molecular spectroscopy. Hence, the objectives of this study were to (1) study cool-season adapted CDC chickpeas molecular structure features and molecular structure changes induced by thermal processing and (2) reveal the association of molecular profiles with nutrient utilization and availability in ruminant systems. It was hypothesized that (1) the molecular intrinsic structure related to nutrient utilization and availability could significantly differ among CDC chickpea varieties and among thermal heat processing methods, and (2) there was an association between molecular structure features of chickpeas and nutrient utilization and availability in the ruminant system.

## 2. Materials and Methods

All cows involved in the present study were cared for in accordance with the guidelines of the Canadian Council on Animal Care [9]. The Animal Use Approval Protocol (19910012) was approved by the Animal Research Ethics Board (AREB) at the University of Saskatchewan (Saskatoon, SK, Canada).

### 2.1. Sample Preparation and Thermal Processing

Chickpea samples from three different varieties were grown in western Canada. Samples were provided by the Crop Development Center (CDC, University of Saskatchewan, Saskatoon, SK, Canada). Three thermal processing methods (microwaving, autoclaving, drying heating) were also applied to each chickpea sample. The detailed growth conditions, sampling procedure, and size were reported previously. All samples (variety study samples and thermal processing samples) were analyzed for (1) chemical composition using AOAC [10] and Van Soest et al. [11] standard procedures; (2) total digestible nutrients (TDN_1x_) and energy values (DE, ME, NE_L3x_, NE_m_, NE_g_) for dairy and beef cattle [12,13]; (3) protein and carbohydrate subfractions using an CNCPS 6.5 system (such as PA1, PA2, PB1, PB2, PC; CA4, CB1, CB2, CB3, CC) [14]; (4) rumen degradation kinetics of nutrients (S, D, U, Kd, RD, RU) [15,16]; (5) intestinal and total track digestibility (such as % dRUP, IDP, TPD) [17]; and (6) truly digestible nutrient supplies to dairy cows using the DVE/OEB system [16,18,19,20] and NRC Dairy [12,13,21]. The detailed material, methods, and results for chemical and nutrition value of the chickpeas have been published previously [22]. These nutrition data were used for this relationship study. For FTIR molecular spectroscopic study, all the samples were ground through a 0.12 mm screen (Retsch ZM 200, Retch Inc., Haan, Germany).

### 2.2. Univariate Molecular Spectral Analysis of Functional Groups Related to Carbohydrates and Proteins

The FTIR analysis was performed using a JASCO-FTIR-4200 spectroscope (JASCO Corp, Tokyo, Japan) at the SRP Feed Research Chair Lab, Department of Animal and Poultry Science at the University of Saskatchewan (Saskatoon, SK, Canada). Molecular structure features of functional groups were analyzed in the mid-infrared region of ca. 4000–800 cm^−1^. Five spectra for each sample were obtained with a resolution of 4 cm^−1^ using the software JASCO Spectra Manager II [3,5,23]. To identify chemical functional groups for carbohydrates and proteins, OMNIC 7.3 software (Spectra Tech, Madison, WI, USA) was used [3,5,23]. For detailed spectroscopic methods, please check our publications [3,5,23].

To detect responses and sensitivity of chemical functional groups to thermal processing methods and compare among varieties, various functional groups and ratios can be tested and analyzed. These functional groups include peaks centered at ca. 1740 (carbonyl C=O ester), ca. 1650 (amide I), ca. 1657 (α-helix), ca. 1630 (β-sheet), ca. 1550 (amide II), ca. 1515 (aromatic compounds of lignin), ca. 1428, ca. 1371 and ca. 1245 (cellulosic compounds), ca. 1025 (nonstructural CHO, starch granules), ca. 1246 (cellulosic material), ca. 1160 (CHO), ca. 1150 (CHO), ca. 1080 (CHO), ca. 930 (CHO), ca. 860 (CHO), ca. 2960 (CH_3_ antisymmetric), ca. 2929 (CH_2_ antisymmetric), ca. 2877 (CH_3_ symmetric) or ca. 2848 cm^−1^ (CH_2_ asymmetric) [6,7,24,25,26].

### 2.3. Association between Molecular Structure Spectral Profiles and Nutrient Metabolic Characteristics of Protein and Carbohydrates

Multiple regression was performed to study molecular spectral features that could explain a variation in chemical profiles, protein and carbohydrate fractions, energy values, in situ rumen degradation, intestinal digestion, and truly absorbable nutrient supply. The detailed methods have been reported previously [3,5,23].

### 2.4. Statistical Analyses

Carbohydrate- and protein-related molecular structure spectral data were analyzed using the “Mixed” procedure of SAS 9.4 (SAS Institute, Inc., Cary, NC, USA). The model used for analysis was Y_ijk_ = μ + T_i_ + S(T_i_) + e_ijk_, where Y_ijk_ is the observation of the dependent variable ijk; μ represents the population mean of the variable; T_i_ means the treatment effect as a fixed effect; and S(T_i_) represents the subsample nested within treatments. In this case, five scans were made per sample, with e_ijk_ representing random errors associated with the observation ijk. The model assumption was tested using the univariate procedure for residual analysis with normal and plot options. All significant analyses were declared at *p* < 0.05 and trends at 0.05 < *p* ≤ 0.10. The treatments were compared by using Tukey’s comparison method.

The associations between molecular structure spectral parameters and chemical composition, rumen degradation, intestinal digestion, and truly absorbable nutrient supply were analyzed using SAS 9.4. Multiple regression study was carried out using the PROC REG procedure. The following model was used: Y = a + b_1_ × x_1_ + b_2_ × x_2_ + … + b_n_ × x_n_. Using STEPWISE for selection criteria: “SLENTRY = 0.05, SLSTAY = 0.05.” Collinearity tests were performed using VIF to eliminate the influence of correlated dependent variables. All variables kept in as predictors were significant at the alpha 0.05 level, and models with an R^2^ greater than 0.65 were selected to report in this study. Univariate procedures were used for residual analysis with normal and plot options.

## 3. Results

### 3.1. Univariate Analysis of Molecular Structure Spectral Profiles in Different Varieties of CDC Chickpeas Grown in Western Canada

In this study, significant differences (*p* < 0.05) were observed in the protein-related spectral profiles among three CDC chickpea varieties: CDC Alma, CDC Cory, and CDC Frontier (Table 1). CDC Frontier and CDC Alma showed higher amide I and II peak heights than CDC Cory (*p* < 0.05). No differences (*p* < 0.05) among the three varieties were observed for cellulosic compounds (CEC) or total carbohydrates (TCHO) related spectral profiles.

Peak ratios of STCHO: TCHO area were higher (*p* < 0.001) in CDC Alma and CDC Frontier (0.32 and 0.34 AU, respectively) than CDC Cory (0.29 AU). The CEC: TCHO area ratio was higher (*p* < 0.001) in CDC Frontier (0.05 AU) than CDC Alma and CDC Cory (0.04 AU for both). In a study from Sun et al. (2018) [23], it was reported that Kabuli and Desi varieties had higher amide I and peak height than barley grain, but no difference in many of the other measured spectral characteristics.

### 3.2. Univariate Analysis of Protein and Carbohydrate Related Molecular Structure Spectral Profiles Using Different Processing Methods

Molecular structure spectral characteristics of CDC chickpeas among thermal processing methods are presented in Table 2. Total amide area was larger (*p* = 0.013) when using autoclave and microwave heating (47.38 and 45.19 AU, respectively) than dry heating (33.06 AU). Amide I peak height was higher (*p* = 0.028) in CDC Alma and CDC Frontier (0.45 AU for both) than CDC Cory (0.36 AU). This indicates that thermal processing induced changes in protein-related spectral profiles, but the sensitivity and response to each processing method differed among treatments. Moreover, the absorbance for STCHO area was higher (*p* = 0.014) with dry heat and microwave treatments. Conversely, CEC area was lower (*p* < 0.01) with dry heat (2.20 AU) than autoclave and microwave heating (3.74 and 3.61 AU, respectively). These results also indicate that thermal processing induced changes in CHO-related molecular spectral profiles, but the sensitivity and response to each processing method differed among treatments.

Results related to peak ratios showed that the height peak ratio of amide I to II was higher (*p* = 0.022) with microwave than dry heat treatment (1.44 vs. 1.16 AU). No significant difference was observed in the other spectral peak ratios analyzed (*p* < 0.05).

### 3.3. Relationship between Protein and Carbohydrates Related Molecular Structure Features and Nutritional and Metabolic Characteristics of Protein and Carbohydrates

The spectral studies showed that several important nutritional parameters could be predicted using protein- and carbohydrate-related molecular spectral features. In rumen degradation kinetics study, the results (Table 3) showed that EDCP and % BDM were associated (*p* < 0.05) with molecular spectral variables related to STCHO peaks and CEC height (R2 = 0.68).

In the chemical profile study (Table 4), ADF_NDF_, hemicellulose, and starch were highly associated (*p* < 0.05) with CEC_STC ratio (R^2^ > 0.63), followed by TC-related peak heights and STC1.

In the chemical and nutrient profile and predicted protein supply studies (Table 5, Table 6 and Table 7), the nutrient supply values were associated (*p* < 0.05) with spectral features of STC1, STC_A, and CEC_A (R^2^ between 0.65 and 0.85).

The results are in partial agreement with Sun et al. (2018) [23], where the authors stated that FTIR molecular spectroscopy, a nondestructive bioanalytical technique, can be used to evaluate true nutrient supply using feed-inherent molecular spectral features for large quantities of feeds in a very short time.

## 4. Discussion

There is very limited published research on spectral features of chickpeas with which to compare our current findings. There is no systematic study on the relationship between molecular structure features of chickpeas and nutrient utilization and availability in ruminant system. There is no study on molecular structure changes induced by thermal processing in cool-season adapted CDC chickpea varieties revealed by advanced vibrational molecular spectroscopy either. Hence, this study provided an insight on specific molecular spectral features that might be associated with nutritional and digestive characteristics of CDC chickpeas.

In this study, heating treatments did impact both nutritional and molecular structure profiles of CDC chickpeas. The response and sensitivity to each thermal processing differed among dry heating, autoclaving, and microwave treatments. This agrees with the effects observed when moisture, pressure, or dry heating is applied to improve the nutritive value of feeds by physical and molecular modifications. Rodriguez-Espinosa et al. [27] indicated that vibrational spectroscopy can also be used for determining the possible alteration of structure during processing.

In this study, rumen degradation kinetics, intestinal digestibility and true nutrient supply to dairy cows were highly associated with molecular structure features in chickpeas. For example, feed milk value (FMV_DVE_) was associated with three spectral variables of STC1, STC_A, and CEC_A (R^2^ = 0.85, *p* < 0.01), and absorbed rumen undegraded protein (ARUP) was associated with spectral variables of STC1, STC_A, and CEC_A (R^2^ = 0.72, RSD = 22.53, *p* < 0.01). Total true protein supply (DVE) to dairy cows and total metabolizable protein (MP) were associated with three spectral variables of STC1, STC_A and CEC_A (R^2^ > 0.70, RSD = 21, *p* < 0.01). The studies conducted by Xin et al. [28] (2014) and Xin and Yu [29] showed that the spectral features are correlated with nutrient values. However, in studies conducted by Xin and Yu [30,31] to compare the alteration of spectral profiles of canola and *Brassica carinata* during microbial digestion, chemical profiles were used to correlate with structural change, which cannot reflect the utilization and digestion condition of the feed.

Molecular spectral analysis is a useful method to associate with nutrient values. The spectral variable analysis showed that with molecular spectroscopic technique-FTIR, the true nutrient supply to dairy cows was associated with a few specific molecular spectral parameters in the vibrational mid-infrared region—ca. 4000–800 cm^−1^.

These results show us the potential of the molecular spectroscopic technique of FTIR in using spectral parameters in the vibrational mid-infrared region to predict the nutrient supply in ruminant systems. The implication of this study is that feed nutritional value is not only related to total chemical composition but also inherent molecular structure. Future NRC models or any other feed evaluation systems could consider feed molecular structure features as an important part of developing modern and improved nutrition models.

## 5. Conclusions

In conclusion, CDC Frontier and CDC Alma showed higher peak heights in protein-related profiles. However, CDC Alma, CDC Cory, and CDC Frontier tended to differ in peak area of cellulosic compound profiles. Area of protein-related molecular spectral profiles differed among dry heat, autoclave, and microwave treatments in amide I and amide II. STCHO peak heights were higher in the 1st and 3rd peaks for dry heat treatment. Greater values were observed in the CEC spectral area in autoclave and microwave treatments (average 3.65 AU) and lower absorbance in dry heat treatment with 2.20 AU. The peak height ratio in the amide I to II area was higher than in the microwave treatment.

Vibrational molecular mid-infrared FTIR spectroscopy was able to reveal different molecular spectral characteristics among the cool-season adapted CDC chickpea varieties and detect molecular structure changes induced by thermal processing (dry heating, autoclaving, and microwave heating). There is an association between molecular structure spectral features of chickpeas and nutrient availability in ruminant systems.

## Figures and Tables

**Table 1:** Cool-Climate Adapted CDC Genotypes of Chickpeas Grown in Western Canada: Protein and carbohydrate related molecular structures spectral profiles (unit: A.U.) of different chickpea varieties using Fourier-transform infrared attenuated total reflectance molecular spectroscopy.

	Chickpea Variety				
Items	CDC Alma	CDC Cory	CDC Frontier	SEM ^a^	*p*-Value
Protein-Related Spectral Profiles ^b^					
Total amide	49.92 ^ab^	39.86 ^b^	50.79 ^a^	3.505	0.021
Amide I area	30.45 ^ab^	24.64 ^b^	30.85 ^a^	2.196	0.038
Amide II area	19.46 ^a^	15.21 ^b^	19.94 ^a^	1.348	<0.001
Amide I peak height	0.45 ^a^	0.36 ^b^	0.45 ^a^	0.030	0.028
Amide II peak height	0.33 ^a^	0.26 ^b^	0.33 ^a^	0.021	0.015
Structural Carbohydrate (STCHO)-Related Spectral Profile					
1st peak height	0.12	0.10	0.12	0.008	0.128
2nd peak height	0.16	0.14	0.16	0.009	0.119
3rd peak height	0.10 ^a^	0.08 ^b^	0.10 ^a^	0.006	0.039
STCHO area	23.74	20.23	23.27	1.295	0.064
Cellulosic Compound (CEC)-Related Spectral Profile					
CEC peak height	0.07	0.06	0.07	0.004	0.141
CEC area	3.48	2.88	3.44	0.222	0.057
Total Carbohydrate (TCHO)-Related Spectral Profile					
1st peak height	0.20	0.19	0.18	0.011	0.612
2nd peak height	0.44	0.40	0.41	0.024	0.424
3rd peak height	0.57	0.56	0.54	0.028	0.597
TCHO area	72.44	69.11	67.15	3.697	0.545
Spectral Peak Ratios					
Amide I: II area	1.56	1.62	1.53	0.036	0.106
Amide I: II height	1.36	1.39	1.36	0.026	0.640
STCHO: TCHO area	0.32 ^a^	0.29 ^b^	0.34 ^a^	0.006	<0.001
CEC: TCHO area	0.04 ^b^	0.04 ^b^	0.05 ^a^	0.001	<0.001
CEC: STCHO area	0.14	0.14	0.14	0.006	0.776

^a^ SEM, standard error of the mean. ^b^ Means with different letters in the same row are significantly different (*p* < 0.05). Multitreatment comparisons using Tukey’s method. STCHO (peak area region and baseline, ca. 1416–1238 cm^−1^); TCHO (peak area region and baseline, ca. 1186–946 cm^−1^); CEC (peak area region and baseline, ca. 1274−1238 cm^−1^). The peak area and the peak height presented in each functional group measurements are expressed in IR absorbance units.

**Table 2:** Cool-Climate Adapted CDC Genotypes of Chickpeas Grown in Western Canada: Protein and carbohydrate molecular structures spectral profiles (A.U. Unit) of different thermal processing methods using Fourier-transform infrared attenuated total reflectance molecular spectroscopy.

	Processing Methods				
Items	Dry Heat	Autoclave	Microwave	SEM ^a^	*p*-Value
Protein Related Spectral Profile ^b^					
Total amide	33.06 ^b^	47.38 ^a^	45.19 ^a^	4.491	0.013
Amide I area	19.71 ^b^	29.88 ^a^	28.19 ^a^	2.952	0.007
Amide II area	13.35 ^b^	17.50 ^a^	13.35 ^b^	1.598	0.043
Amide I peak height	0.30 ^b^	0.43 ^a^	0.41 ^a^	0.042	0.015
Amide II peak height	0.25	0.31	0.29	0.025	0.128
Structural carbohydrate (STCHO)-Related Spectral Profile					
1st peak height	0.07 ^b^	0.10 ^ab^	0.11 ^a^	0.011	0.011
2nd peak height	0.14	0.14	0.15	0.008	0.424
3rd peak height	0.06 ^b^	0.10 ^a^	0.10 ^a^	0.010	0.003
STCHO area	23.07 ^a^	21.10 ^ab^	23.07 ^a^	2.352	0.014
Cellulosic compound (CEC)-Related Spectral Profile					
CEC peak height	0.05 ^b^	0.07 ^a^	0.07 ^a^	0.006	0.005
CEC area	2.20 ^b^	3.74 ^a^	3.61 ^a^	0.348	<0.001
Total carbohydrate (TCHO)-Related Spectral Profile					
1st peak height	0.14 ^b^	0.18 ^a^	0.21 ^a^	0.016	<0.001
2nd peak height	0.30 ^b^	0.40 ^ab^	0.45 ^a^	0.044	<0.001
3rd peak height	0.35 ^b^	0.58 ^a^	0.62 ^a^	0.083	<0.001
TCHO area	42.06 ^b^	69.14 ^a^	76.27 ^a^	10.525	<0.001
Peak Ratios					
Amide I: II area	1.36	1.71	1.36	0.133	0.152
Amide I: II height	1.16 ^b^	1.37 ^ab^	1.44 ^a^	0.071	0.022
STCHO: TCHO area	0.30	0.30	0.25	0.065	0.805
CEC: TCHO area	0.07	0.05	0.04	0.029	0.802
CEC: STCHO area	0.19	0.17	0.15	0.035	0.801

^a^ SEM, standard error of the mean. ^b^ Means with different letters in the same row are significantly different (*p* < 0.05). Multitreatment comparisons using Tukey’s method. STCHO (peak area region and baseline, ca. 1416–1238 cm^−1^); TCHO (peak area region and baseline, ca. 1186–946 cm^−1^); CEC (peak area region and baseline, ca. 1274−1238 cm^−1^). The peak area and the peak height presented in each functional group measurements are expressed in IR absorbance units.

**Table 3:** Multiple Regression Analysis to Study Association between Protein Spectral Parameters and Protein-Related Nutrition Values of Cool-Season Adapted CDC Chickpeas.

Variable (y)	Variable Selection (*p* < 0.05)	$Y=a+b_{1}\times x_{1}+b_{2}\times x_{2}+\ldots +b_{n}\times x_{n}$	R^2^	RSD	*p* Value
Protein profiles					
CP (%DM)	HAII	CP (%DM) = 29.63 HAII + 12.66	0.44	1.63	0.004
ADICP (%CP)	HAII	ADICP (%CP) = 1.033 HAII − 0.198	0.21	0.09	0.050
PC (%CP)	HAII	PC (%CP) = 1.03 HAII − 0.20	0.22	0.09	0.059
ADICP (%DM)	HAI, HAII	ADICP (%DM) = 0.22 HAII − 0.04	0.39	0.02	0.030
Protein sub-fractions					
TP (%CP)	HAII	TP (%CP) = −1.03 HAII + 100.20	0.22	0.09	0.055
TotRDP (%CP)	HAII	TotRDP (CP%) = 29.36 HAII + 12.72	0.44	1.64	0.004
RUPC (%CP)	HAII	RUPC (CP%) = 0.28 HAII − 0.06	0.32	0.02	0.018
TotRUP (%CP)	HAII	TotRUP (CP%) = 29.63 HAII + 12.67	0.44	1.63	0.003
tdCP (%CP)	HAII	TdCP (%CP) = 30.00 HAII + 12.54	0.45	1.62	0.003
CP (%CP)	AII	CPg (%CP) = 2.30 AII + 160.63	0.22	17.59	0.057
CP Degradation					
BCP (g/kg DM)	HAII	BCP(g/kg DM) = 585.74 HAII − 105.41	0.18	61.93	0.093
D (%)	HAI_AII	D(%) = 47.09 HAI_AII − 4.53	0.29	10.64	0.026
U (%)	AAI_AII	U(%) = −36.52 AAI_AII + 70.17	0.23	10.95	0.052
EDCP (%)	STC1, STC3	EDCP (%) = −289.54 STC1 + 215.88 STC3 + 21.97	0.68	6.62	0.001

Notes: TotRDP; total rumen degraded protein; RUPC: rumen undegradable protein; TotRUP: total rumen undegradable protein; tdCP: truly digestible crude protein; BCP: rumen bypass feed crude protein (DVE/OEB system); D: degradable fraction; U: rumen undegradable fraction; EDNDF: effective degraded neutral detergent fiber. HAI_AII: amide I to amide II peak height ratio; RSD: residual standard deviation; R2: coefficient of determination. All variables left in the final model were significant at the 0.05 level.

**Table 4:** Multiple Regression Analysis to Study Association between Protein Spectral Parameters and Truly Absorbed Protein, Intestinal Protein Degradation, Rumen Degradation Parameters of Cool-Season Adapted CDC Chickpeas.

Variable (y)	Variable Selection (*p* < 0.05)	$Y=a+b_{1}\times x_{1}+b_{2}\times x_{2}+\ldots +b_{n}\times x_{n}$	R^2^	RSD	*p* Value
DVE-OEB model					
DVE (g/kg DM)	HAII	DVE (g/kg DM) = 342.61 HAII + 26.46	0.20	33.23	0.070
MREE (g/kg DM)	HAII	MREE (% CP) = −81.22 HAII + 154.49	0.17	8.74	0.098
DVME (g/kg DM)	HAII	DVME (% CP) = −51.74 HAII + 99.75	0.17	5.57	0.098
DVBE (g/kg DM)	HAII	DVBE (g/kg DM) = −72.58 HAII + 393.88	0.20	38.75	0.073
FMV_DVE_ (% CP)	HAII	FMV_DVE_ (% CP) = 7.04 HAII + 0.16	0.20	0.70	0.077
NRC Model					
MP (g/kg DM)	HAII	MP = 347.340 HAII + 7.80	0.20	34.65	0.078
FMV (g/kg DM)	HAII	FMV_NRC_ = 7.05 HAII + 0.16	0.19	0.70	0.077
Truly digestible nutrient supply to dairy cows					
ARUP (g/kg DM)	HAII	ARUP(%CP) = 354.80 HAII − 65.37	0.20	37.91	0.073

Notes: DVE: truly digested protein in the small intestine; OEB: degraded protein balance; MREE: microbial protein synthesized in the rumen based on available energy; DVME: truly absorbed rumen synthesized microbial protein in the small intestine; DVBE: truly absorbed bypass feed protein in the small intestine; MP: metabolizable protein (NRC Dairy model); FMV: feed milk value; ARUP: truly absorbed rumen undegraded protein in the small intestine (NRC Dairy model). HAII: amide II peak height; RSD: residual standard deviation; R^2^: coefficient of determination. All variables left in the final model were significant at the 0.05 level.

**Table 5:** Multiple Regression Analysis to Study Associations between Carbohydrate Spectral Parameters and Chemical and Nutrient Profiles, Truly Absorbed Protein, Intestinal Protein Degradation, Rumen Degradation Parameters of Cool-Season Adapted CDC Chickpeas.

Variable (y)	Variable Selection (*p* < 0.05)	$Y=a+b_{1}\times x_{1}+b_{2}\times x_{2}+\ldots +b_{n}\times x_{n}$	R^2^	RSD	*p* Value
Energy Values					
DE_p3X_	TC1, TC3	DEp_3x_ = −7.52 TC1 + 4.31 TC3 + 2.83	0.44	0.18	0.022
ME_Beef_	TC3, TC1	ME_beef_ = 4.312 TC3 − 7.92 TC1 + 2.49	0.43	0.19	0.244
Degradation Kinetics					
U	STC2	U = 137.86 STC2 − 12.98	0.44	2.81	0.005
%BDM	STC2, CEC_H	%BDM =512.02 STC2 − 897.69 CEC_H + 73.03	0.68	7.12	0.002
Rumen CHO Degradation					
EDCP	STC_TC	EDCP = −50.86 stc_tc − 50.86	0.51	13.45	0.001
Truly Digestible Nutrients					
tdNFC	CEC_H, TC1	tdNFC = −447.16 cec_H + 145.331 tc1 + 64.80	0.67	2.88	0.008
tdNDF	CEC_STC	tdNFC = 52.22 cec_stc + 0.43	0.23	2.47	0.059

Notes: DEp3×: digestible energy at a production level (3× maintenance); ME_Beef_: metabolizable energy beef; U: rumen undegradable fraction; EDCP: effective degraded crude protein; tdNDF: truly digestible neutral detergent fiber; tdNFC: truly digestible non-fiber carbohydrate; TC1, total carbohydrate first peak height; STC2, structural carbohydrate second peak height; TC3, total carbohydrate third peak height; CEC_Height: cellulosic compound peak height; CEC_STC: cellulosic compound structural carbohydrates; RSD: residual standard deviation; R^2^: coefficient of determination. All variables left in the final model were significant at the 0.05 level.

**Table 6:** Multiple Regression Analysis to Study Associations between Carbohydrate Spectral Parameters and Carbohydrate Chemical Profiles and Nutrition Values of Cool-Season Adapted CDC Chickpeas.

Variable (y)	Variable Selection (*p* < 0.05)	$Y=a+b_{1}\times x_{1}+b_{2}\times x_{2}+\ldots +b_{n}\times x_{n}$	R^2^	RSD	*p* Value
Basic Nutrient Profiles (%DM)					
CHO	STC3	CHO = −102.22 stc3 − 83.75	0.40	1.75	0.008
NFC_CHO_	CEC_STC	NFC_cho_ = −16.73 cec_stc + 109.62	0.31	4.56	0.026
NDF	CEC_STC	NDF = 113.0 cec_stc − 2.87	0.25	5.13	0.049
iNDF	CEC_TC	iNDF = 79.31 cec_tc − 3.04	0.30	0.67	0.029
ADF	STC1	ADF = 130.64 stc1 − 9.98	0.31	2.76	0.026
ADF_NDF_	STC1, CEC_STC	ADF_NDF_ = 923.25 stc1 − 420.40 − 4.25	0.66	11.95	0.009
ADL_NDF_	TC3	ADL_NDF_ = −20.14 tc3 + 1235	0.19	0.95	0.881
Hemicellulose	TC1, CEC_STC	Hemicellulose = 160.56 cec_stc − 153.79 tc1 + 13.95	0.63	3.50	0.002
Cellulose	STC1	Cellulose = 13.23 stc1 − 8.76	0.33	2.27	0.020
Starch	TC1, TC3, CEC_STC	Starch = 118.31 tc1 − 77.31 tc3 − 101.88 cec_stc	0.66	344	0.004
Sugar	CEC_TC,	Sugar = −161.63 cec_tc + 21.15	0.30	1.36	0.028
Sugar_NFC_	TC3	Sugar_NFC_ = 24.47 tc3 + 5.93	2.02	0.25	0.049
Carbohydrate Subfractions (%DM)					
CA4_CHO_	CEC_TC	CA4_CHO_ = −161.63 cec_tc + 21.14	0.30	1.36	0.028
CB1_CHO_	CEC_STC, TC1, TC3	CB1_CHO_ = −101.88 cec_stc + 118.31 tc1 − 77.31 tc3 + 84.28	0.66	3.44	0.004
CB3_CHO_	CEC_STC	CB3_CHO_ = 113.74 cec_stc − 1.01	0.36	4.93	0.042
RDCA_4_	CEC_TC	RDCA_4_ = −161.63 cec_tc + 21.15	0.30	1.36	0.280
RDCB_1_	TC1, TC3, CEC_STC	RDCB_1_ = 118.31 tc1 − 77.31 tc3 − 101.88 cec_stc + 84.29	0.66	3.44	0.006
RDCB_3_	STC3, TC1	RDCB_3_ = −153.89 stc3 + 49.14 tc1 + 57.94	0.69	1.17	0.000
RUCA_4_	CEC_TC	RUCA_4_ = −31.94 cec_tc + 3.80	0.33	0.25	0.020
RUCB_1_	TC3, CEC_STC	RUCB_1_ = −9.84 tc3 − 17.58 cec_stc + 16.41	0.56	0.72	0.005
RUCB_3_	CEC_STC	38.96 cec_stc + 0.052	0.23	1.84	0.059

Notes: CHO: carbohydrates; NFC: non-fiber carbohydrate; NDF: neutral detergent fiber; ADF: acid detergent fiber; ADL: acid detergent lignin; CA4 = sugar (rapidly degradable carbohydrate fraction); CB1 = starch (intermediately degradable carbohydrate fraction); CB3 = digestible fiber (available neutral detergent fiber or slowly degradable carbohydrate fraction); TC3: total carbohydrate third peak height; STC4: structural carbohydrate third peak height; CEC_STC: cellulosic compound structural carbohydrates; RSD: residual standard deviation; R2: coefficient of determination. All variables left in the final model were significant at the 0.05 level.

**Table 7:** Multiple Regression Analysis to Study Associations between Carbohydrate Spectral Parameters and Truly Absorbed Protein, Intestinal Protein Degradation and Protein Rumen Degradation Kinetics of Cool-Season Adapted CDC Chickpeas.

Variable (y)	Variable Selection (*p* < 0.05)	$Y=a+b_{1}\times x_{1}+b_{2}\times x_{2}+\ldots +b_{n}\times x_{n}$	R^2^	RSD	*p* Value
Truly Digestible Nutrient Supply to Dairy Cows					
MREE	STC1, STC_A, CEC_A	MREE = −338.54 stc1 − 2.11 stc_A + 12.77 cec_A + 171.86	0.70	5.72	0.001
DVME	STC1, STC_A, CEC_A	DVME = −215.70 stc1 − 1.35 stc_A + 8.15 cec_A + 109.56	0.70	3.65	0.001
DVBE	STC1, STC_A, CEC_A	DVBE = 1563.44 stc1 + 9.85 stc_A − 54.42 cec_A − 155.75	0.72	25.01	0.001
MREN	STC1, CEC_STC	MREN = −1776.54 stc1 + 1953.25 cec_stc + 31.49	0.65	40.94	0.001
FMV_DVE_	STC1, STC_A, CEC_A	FMV_DVE_ = 28.59 stc1 + 0.18 stc_A − 1.011 cec_A − 1.33	0.85	0.44	0.009
FMV_NRC_	STC1, STC_A, CEC_A	FMV_NRC_ = 28.59 stc1 + 0.18 stc_A − 1.01 cec_A − 1.33	0.73	0.44	0.009
ARUP	STC1, STC_A, CEC_A	ARUP = 1408.57 stc1 + 8.88 stc_A − 49.03 cec_A − 140.30	0.72	22.53	0.001
AECP	CEC_H, TC_A	AECP = −2.66 cec_H + 0.01 tc_A + 3.99	0.36	0.04	0.050
MCP_RDP_	STC_TC	MCP_RDP_ = 323.93 stc_tc − 43.24	0.51	11.43	0.001
Degraded protein balance (OEB) and Total true protein supply (DVE) to dairy cows					
DVE	STC1, STC_A, CEC_A	DVE = 1346.82 stc1 + 8.51 stc_A − 46.22 cec_A − 47.02	0.72	21.45	0.001
OEB	STC1, CEC_STC	OEB = −1416.15 stc1 + 1690.59 cec_stc − 99.12	0.64	36.28	0.001
Degraded protein balance (DPB) and Total metabolizable protein supply (MP) to dairy cows					
MP	STC1, STC_A, CEC_A	MP = 1407.09 sct1 + 8.82 stc_A − 49.70 cec_A − 65.68	0.73	21.64	0.009
DPB	STC_TC	DPB = 395.34 stc_tc − 178.24	0.55	12.93	0.001

Notes: MREE: microbial protein synthesized in the rumen based on available energy; DVME: truly absorbed rumen synthesized microbial protein in the small intestine; DVBE: truly absorbed bypass feed protein in the small intestine; DVE: total truly digested protein in the small intestine (DVE/OEB system); STC4: structural carbohydrate fourth peak height; CEC_AREA: cellulosic compound peak area; TC4: total carbohydrate fourth peak height; STC3: structural carbohydrate third peak height; TC3: total carbohydrate third peak height; STC1: structural carbohydrate first peak height; RSD: residual standard deviation; R2: coefficient of determination. All variables left in the final model were significant at the 0.05 level.

## Data Availability

The data that support the findings of this study are available from the corresponding author upon reasonable request.
